# SIRT3 inhibits cardiac hypertrophy by regulating PARP-1 activity

**DOI:** 10.18632/aging.102862

**Published:** 2020-03-04

**Authors:** Xiaojun Feng, Yanan Wang, Wenxu Chen, Suowen Xu, Lingli Li, Yadi Geng, Aizong Shen, Hui Gao, Lei Zhang, Sheng Liu

**Affiliations:** 1 The First Affiliated Hospital of USTC, Division of Life Sciences and Medicine, University of Science and Technology of China, Hefei, Anhui, PR. China; 2 Department of Pharmaceutics, College of Pharmacy, Anhui University of Chinese Medicine, Hefei, Anhui, PR. China; 3 Aab Cardiovascular Research Institute, University of Rochester, West Henrietta, NY 14586, USA; 4 Anhui Provincial Cardiovascular Institute, Hefei, Anhui, PR. China; 5 Department of Pharmacology, School of Medicine, Shaoxing University, Shaoxing, PR. China; 6 Equal contribution

**Keywords:** cardiac hypertrophy, SIRT3, PARP-1, acetylation, ribosylation

## Abstract

Sirtuin 3 (SIRT3) is a type III histone deacetylase that inhibits cardiac hypertrophy. It is mainly localized in the mitochondria and is thus implicated in mitochondrial metabolism. Recent studies have shown that SIRT3 can also accumulate in the nuclear under stressed conditions, and participated in histone deacetylation of target proteins. Poly [ADP-ribose] polymerase 1 (PARP-1) functions as an important PARP isoform that was involved in cardiac hypertrophy. Our experiments showed that SIRT3 accumulated in the nuclear of cardiomyocytes treated with isoproterenol or SIRT3 overexpression. Moreover, overexpression of SIRT3 by adenovirus inhibited the expression of cardiac hypertrophic genes-ANF and BNP, as well as abrogating PARP-1 activation induced by isoproterenol or phenylephrine. In addition, co-immunoprecipitation experiments revealed that SIRT3 could interact with PARP-1, and overexpression of SIRT3 could decrease the acetylation level of PARP-1. Our results indicate that SIRT3 exerts protective effects against cardiac hypertrophy by reducing the level of acetylation and activity of PARP-1, thus providing novel mechanistic insights into SIRT3-mediated cardiprotective actions.

## INTRODUCTION

Cardiac hypertrophy is an important pathological stage in the induction of chronic heart failure by pressure overload [1–5]. Initial cardiac hypertrophy is a compensatory process that is beneficial for maintaining myocardial contractility and enhancing cardiac function. However, persistent cardiac hypertrophy reduces myocardial compliance, which is insufficient to maintain the body's requirements for blood pumping. The heart function will gradually deteriorate and eventually develop into heart failure [6]. Prevention of ventricular remodeling can slow down the progression of cardiac insufficiency, prolong the survival time of patients, and improve their prognosis [7–10]. Therefore, studying the pathogenesis of cardiac hypertrophy has great significance for the treatment of heart failure.

The poly ADP-ribose (PAR) polymerase (PARP) family is an important enzyme essential for DNA damage repair in eukaryotic cells [2]. PARP-1 is the first identified member of the PARP family, which is activated by DNA damage and has DNA repair capabilities. The basic structure of PARP-1 is highly conserved in eukaryotic cells, and its catalytic domain also exhibits high homology between different species [11, 12]. In addition to being activated by DNA damage, several types of post-translational modifications can also affect the activity of PARP-1. For example, protein kinase C (PKC) and DNA protein kinase (DNA-PK) can phosphorylate PARP-1, interfere with its DNA binding ability, and inhibit its activity [13, 14]. However, JNK1 and ERK1/2 phosphorylate and activate PARP-1 [15, 16]. Acetylation can also affect the function of PARP-1. For example, histone acetyl-transferase P300 can acetylate PARP-1, and acetylated PARP-1 can promote the transcriptional activity of nuclear factor kappa B (NF-κB) [17]. Previous studies have shown that SIRT1 deacetylates PARP-1 and inhibiting its activity in cardiomyocytes [18]. Our previous studies showed that PARP-1 activity was up-regulated with increasing isoproterenol (ISO) stimulation time and reached its peak at 24 h. Moreover, ISO stimulation significantly up-regulated the acetylation level of PARP-1 [19].

In recent years, PARP-1 has been extensively studied in cardiovascular diseases. In an animal model of pathological hypertrophy, PARP-1 activity was significantly up-regulated [12, 20]. PARP-1 inhibitor and PARP-1 knockdown can inhibit angiotensin II (AngII)-induced cardiac hypertrophy [11]. In addition, studies have shown that treatment with PARP-1 inhibitor (L-2286) can significantly reduce cardiac hypertrophy caused by isoproterenol (ISO) [21]. PARP-1 activation induces cardiac hypertrophy mainly by catalyzing the transfer of ADP-ribose from nicotinamide adenine dinucleotide (NAD^+^) to its target protein, consuming intracellular NAD^+^, impairing energy metabolism, and causing decreased activity of type III histone deacetylase (SIRTs). The decrease of SIRT3 and SIRT6 activity in the SIRT family can cause significant cardiac hypertrophy phenotype [22, 23]. In addition, PARP-1 can increase chromatin remodeling or directly promote the transcriptional activities of transcription factors (such as AP-1 and NF-κB) to upregulate the expression of intracellular adhesion molecule 1 (ICAM-1), and various cytokines and chemokines, which could contribute to the inflammatory mechanisms that promote cardiac hypertrophy [2, 12, 17].

The SIRTs family is a type III type histone deacetylase whose activity is dependent on NAD^+^ [24]. The SIRTs family has seven members: SIRT1~7, which all contain a conserved catalytic core region (consisting of approximately 275 amino acids) [25]. The subtypes of the SIRTs family are widely distributed in tissues, but there are large differences in their intracellular distribution [26]. SIRTs are important therapeutic targets in treating cardiovascular diseases, such as cardiac hypertrophy and atherosclerosis [27, 28]. Among them, SIRT1 and SIRT2 are distributed in both cytoplasm and nuclear, SIRT6 and SIRT7 are mainly located in the nuclear, while SIRT3, SIRT4 and SIRT5 are mainly located in mitochondria. Although SIRT3 is thought to be present in mitochondria, the long fragment of SIRT3 (44 KDa) is able to accumulate in the nuclear under stress [29] (two types of human SIRT3 protein: 44 KDa and 28 KDa, respectively, while rats only have 28 KDa SIRT3 protein).

SIRT3 decreases mitochondrial lysine acetylation levels [30], thereby promoting antioxidant effects and improving mitochondrial function [31, 32]. SIRT3 is involved in the development of many cardiovascular diseases, from cardiac hypertrophy to dilated cardiomyopathy, heart failure, and atherosclerosis [33]. For example, overexpression of SIRT3 can deacetylate and activate superoxide dismutase 2 (SOD2) to reduce intracellular reactive oxygen species (ROS), which in turn improves atherosclerosis [34]. SIRT3 also deacetylates and activate Foxo3a to inhibit cardiac hypertrophy [23]. In addition, overexpression of SIRT3 or exogenous administration of NAD^+^ can inhibit cardiac hypertrophy in mice [31]. However, no report is available as to whether SIRT3 can interact and deacetylate PARP-1 and thereby ameliorating cardiac hypertrophy.

Based on the fact that both SIRT3 and PARP-1 consume NAD^+^ to exert their functions, we hypothesized that SIRT3 could inhibit cardiac hypertrophy by repressing PARP-1 activation in cardiomyocytes.

## RESULTS

### Overexpression of SIRT3 inhibits ISO or PE-induced cardiomyocyte hypertrophy

**Figure 1:**
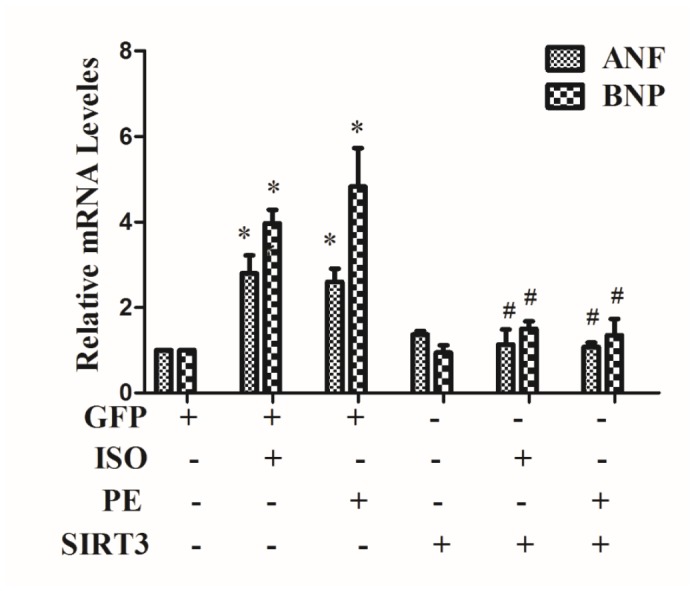
**SIRT3 overexpression inhibits the expression of mRNA levels of ANF and BNP.** In H9c2 cells, Ad-GFP or Ad-SIRT3 were transfected and then stimulated with 10 uM ISO or 100 uM PE for 24 h. RNA was extracted and mRNA expression of ANF and BNP was detected by qRT-PCR. Data were presented as means±SE. *P<0.05 versus control or GFP group, ^#^*P*<0.05 versus GFP treated with ISO or PE, *n*=4 independent experiments.

In H9c2 cells, 10 μM ISO or 100 μM phenylephrine (PE) were used to treat cardiomyocytes for 24 h, using mRNA expressions of cardiac hypertrophic genes ANF and BNP as indicators of cardiomyocyte hypertrophy. The results showed that both ISO and PE treatments significantly up-regulated mRNA expression of ANF and BNP (Figure 1).

In addition, in cardiomyocytes, SIRT3 was overexpressed by Ad-SIRT3, and the inhibitory effect of Ad-SIRT3 on ISO or PE-induced cardiomyocyte hypertrophy was confirmed by detecting hypertrophic genes. The results showed that overexpression of SIRT3 reversed the upregulation of hypertrophic genes by ISO or PE (Figure 1).

### SIRT3 expression and localization in ISO-induced cardiac hypertrophy model

**Figure 2:**
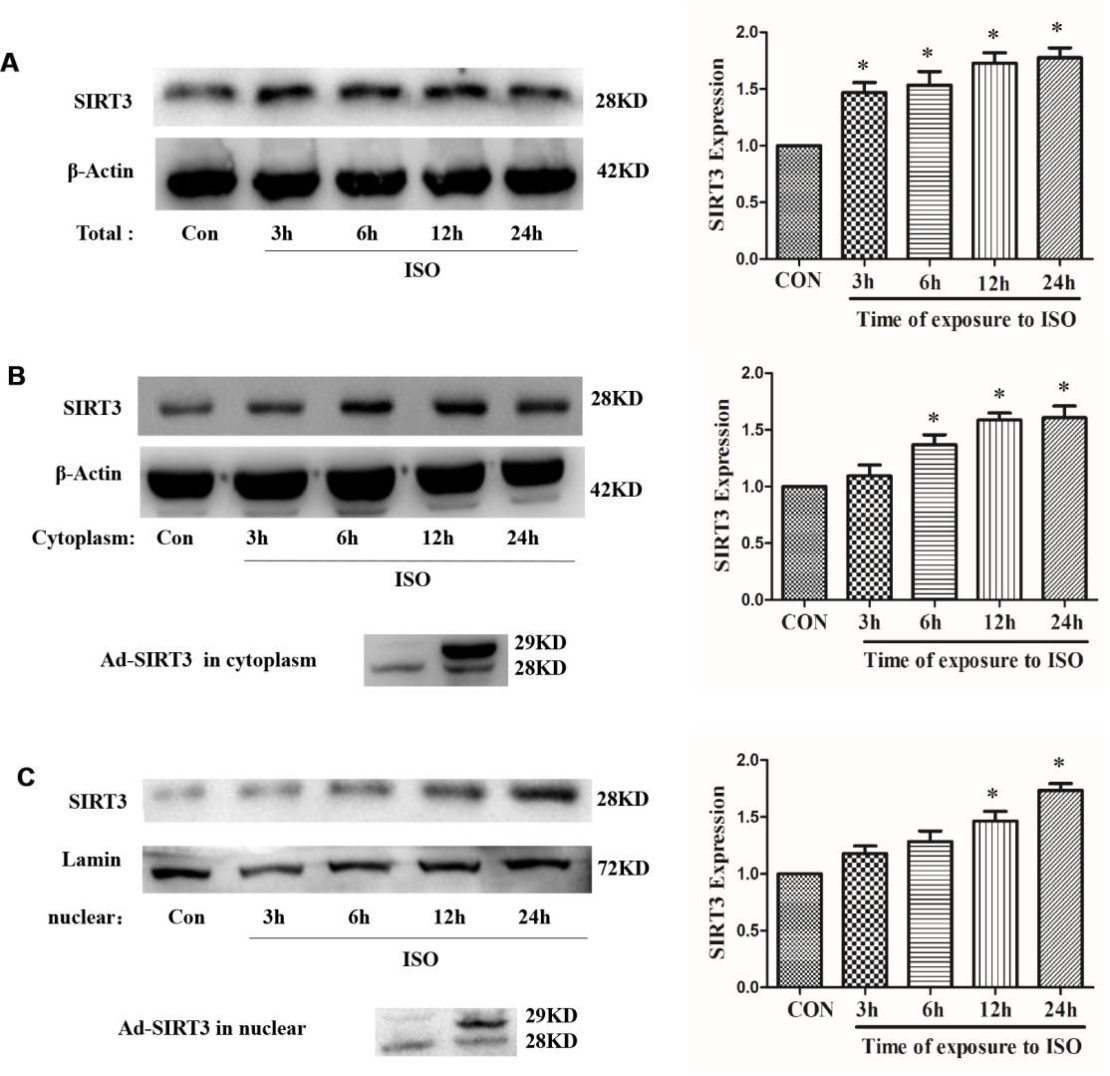
**Distribution of SIRT3 in cytoplasm and nuclear of cardiomyocytes.** In H9c2 cells, Ad-GFP or Ad-SIRT3 were transfected, or 10 μM ISO stimulated for 3-24 h. Total, cytoplasm and nuclear fraction of protein were extracted, and the protein expressions of SIRT3 were detected in total (**A**), cytoplasm (**B**) and nuclear (**C**) by western blot. Data were presented as means±SE. *P<0.05 versus control group, *n*=4 independent experiments.

In H9c2 cells, after treatment with 10 μM ISO for 3-24 h, total protein, cytoplasmic protein and nuclear protein were extracted, and the total protein expression and distribution in cytoplasm of SIRT3 were detected by western blot. The results showed that 10 μM ISO up-regulated the total protein expression of SIRT3 and also increased the distribution of SIRT3 in the cytoplasm and nuclear (Figure 2).

In addition, SIRT3 protein was overexpressed by SIRT3 adenovirus (Ad-SIRT3) in H9c2 cells, and cytosolic protein and nuclear protein were extracted after 24 h. The results showed that Ad-SIRT3 transfection increased the distribution of SIRT3 in cytoplasm and nuclear (Figure 2). Furthermore, in neonatal rat cardiomyocytes, SIRT3 protein was overexpressed with SIRT3-Flag or SIRT3-EGFP plasmid, and a distinct SIRT3 overexpression band was also detected in cytoplasm and nuclear (Supplementary Figure 2).

For *in vivo* studies, SD rats were injected subcutaneously with ISO (1.5 mg/kg/d) for 7 consecutive days. Echocardiography and H&E staining confirmed that the hearts of rats treated with ISO were significantly larger than those of control animals receiving saline and showed typical hypertrophic changes (Supplementary Table 2 and Supplementary Figure 1). Furthermore, ISO increased HW/BW ratio and mRNA expression of hypertrophic genes (ANF and BNP) (Supplementary Figure 1).

**Figure 3:**
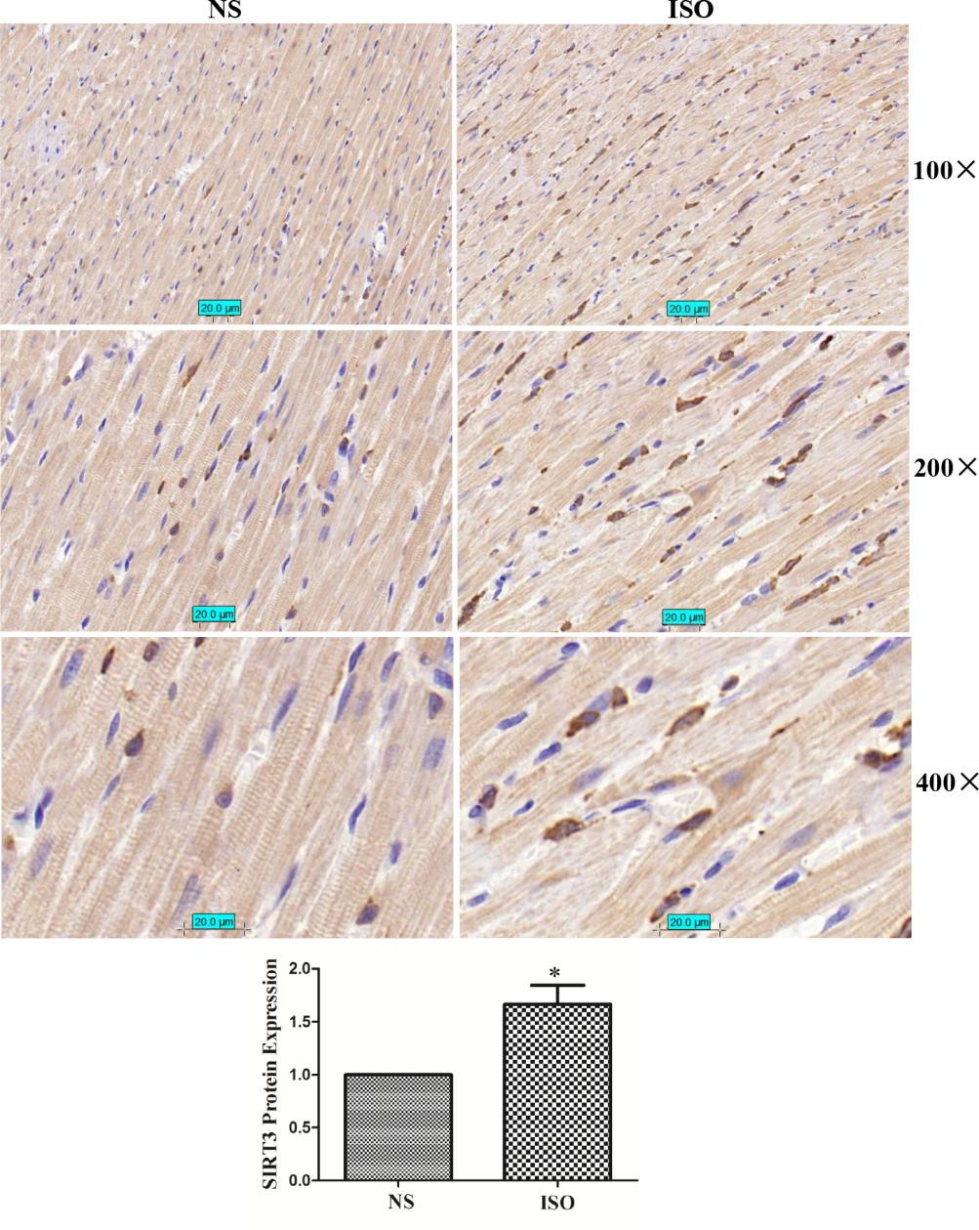
**SIRT3 protein immunohistochemistry in rat heart tissues of NS group and ISO group**. SD rats of ISO group were subjected to subcutaneous injections of 1.5 mg/kg/d isoproterenol for 7 d. The control group was given the same dose of saline. We stained SIRT3 protein with SIRT3 antibody and stained the nuclear with DAPI. Brown represents SIRT3 protein and blue represents nuclear. Representative images out of 4 independent experiments were shown.

**Figure 4:**
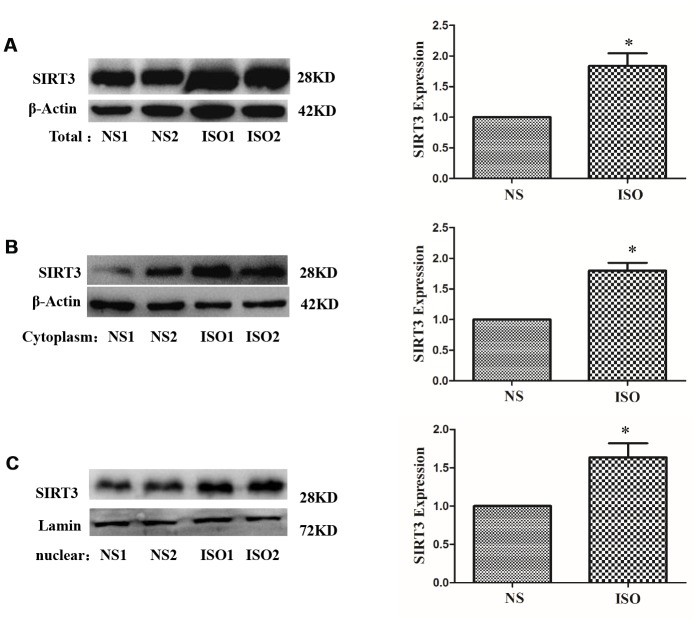
**Distribution of SIRT3 in cytoplasm and nuclear of SD rat heart tissues.** SD rats of ISO group were subjected to subcutaneous injections of 1.5 mg/kg/d isoproterenol for 7 d. The control group was given the same dose of saline. Total, cytoplasm and nuclear fraction of protein were extracted, and the protein expressions of SIRT3 were detected in total (**A**), cytoplasm (**B**) and nuclear (**C**) by western blot. Data were presented as means±SE. *P<0.05 versus control group, *n*=4 independent experiments.

In the ISO-induced SD rat cardiac hypertrophy model, the results of immunohistochemistry showed that the total expression of SIRT3 protein and the distribution in the cytoplasm and nuclear were significantly increased (Figure 3). The total protein, cytoplasmic protein and nuclear protein of SD rat heart tissues were extracted and detected by western blot. The results showed that the total expression of SIRT3 and the distribution in the cytoplasm and nuclear were also significantly increased in the heart tissue of the ISO group (Figure 4).

### SIRT3 overexpression inhibits ISO or PE-induced upregulation of PARP-1 activity

**Figure 5:**
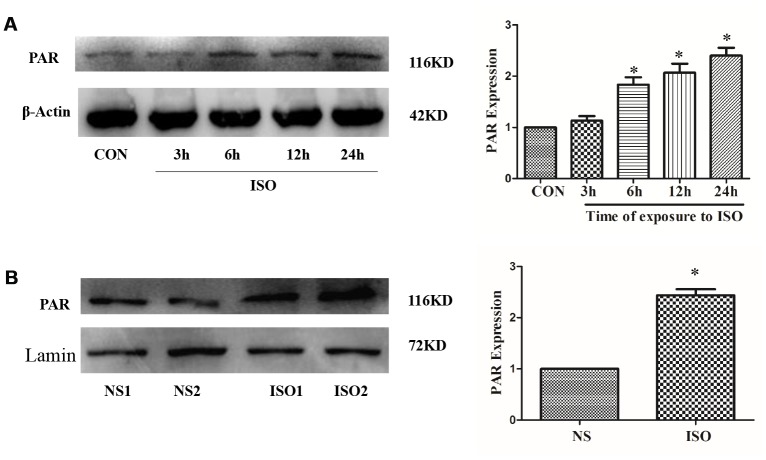
**ISO upregulates PARP-1 activity**. H9c2 cells were treated with 10 μM ISO for 3-24 h and SD rats were subjected to subcutaneous injections of 1.5 mg/kg/d isoproterenol for 7 d. The H9c2 cells protein (**A**) and SD rats’ heart tissues protein (**B**) were extracted after the above treatment. Western blot was used to detect PARP-1 activity. Data were presented as means±SE. *P<0.05 versus CON or NS group, *n*=4 independent experiments.

Our previous studies have shown that PARP-1 activity was significantly up-regulated in cardiac hypertrophy, while inhibition of PARP-1 activity can significantly inhibit cardiac hypertrophy [19, 35]. In H9c2 cardiomyocytes, PARP-1 activity was detected by anti-PAR antibody after 3-24 h stimulation with 10 μM ISO. The result showed that 10 μM ISO significantly up-regulated PAPR-1 activity and was most evident at 24 h (Figure 5A). In addition, in the cardiac hypertrophy model of ISO-induced SD rats, the activity of PARP-1 was also significantly up-regulated compared with the control group (Figure 5B).

**Figure 6:**
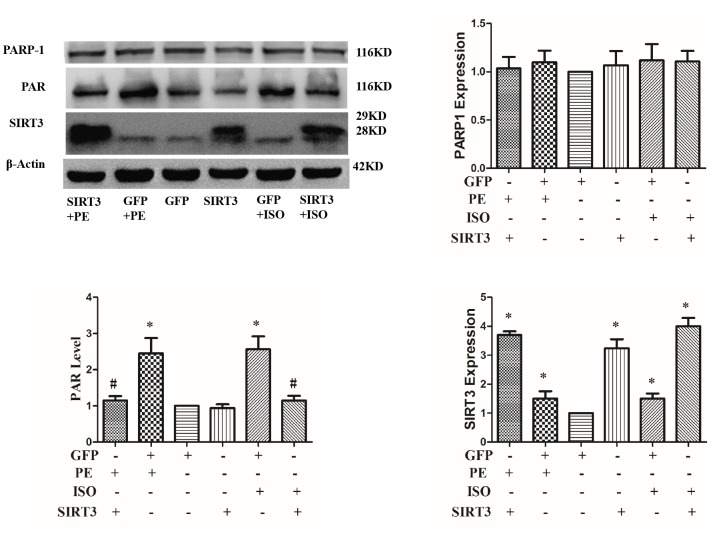
**SIRT3 overexpression inhibits ISO or PE-induced upregulation of PARP-1 activity.** In H9c2 cells, Ad-GFP or Ad-SIRT3 were transfected and then stimulated with 10 μM ISO or 100 μM PE for 24 h. Western blot was used to detect PARP-1, SIRT3 protein expression and PARP-1 activity. Data were presented as means±SE. *P<0.05 versus GFP group, ^#^*P*<0.05 versus GFP treated with ISO or PE, *n*=4 independent experiments.

To investigate whether SIRT3 inhibits cardiac hypertrophy by inhibiting PARP-1 activity, we overexpressed SIRT3 in cardiomyocytes with Ad-SIRT3, then stimulate with ISO or PE for 24 h, and detect PARP-1 activity changes with anti-PAR antibody. The results showed that ISO or PE significantly up-regulated PARP-1 activity, while SIRT3 overexpression obviously inhibited the up-regulation of PARP-1 activity. This result suggests that SIRT3 inhibition of cardiac hypertrophy may be through inhibition of PARP-1 activity (Figure 6).

### SIRT3 interacts with and deacetylates PARP-1

**Figure 7:**
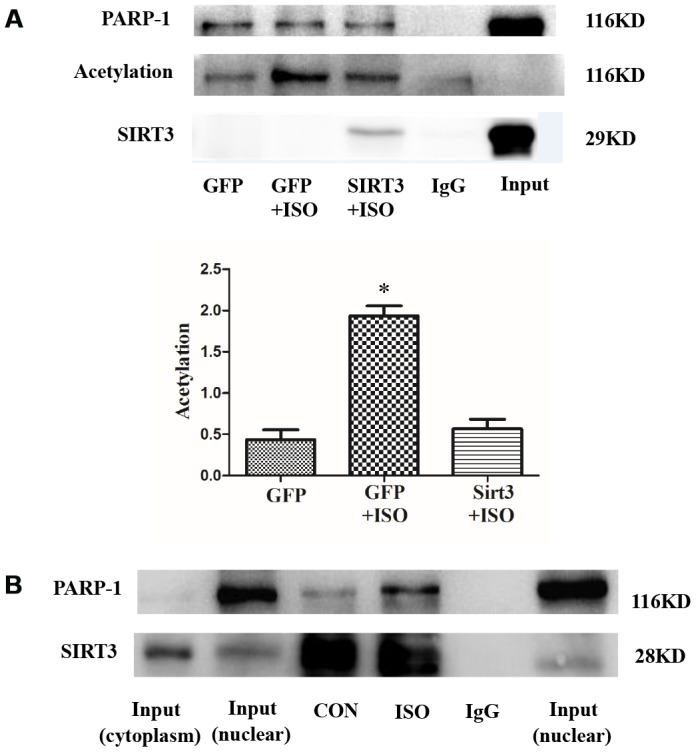
**SIRT3 interacts with and deacetylates PARP-1 in cardiomyocytes**. In H9c2 cells, Ad-GFP or Ad-SIRT3 were transfected and then stimulated with 10 μM ISO for 24 h. PARP-1 was precipitated using PARP-1 antibody and detected with acetylated antibody, PARP-1 antibody, and SIRT3 antibody (**A**). In H9c2 cells, nuclear protein was extracted after 24 h stimulation with ISO. Nuclear SIRT3 protein was precipitated with SIRT3 antibody, and corresponding protein expression was detected with PARP-1 antibody and SIRT3 antibody (**B**). Data were presented as means±SE. *P<0.05 versus GFP group, ^#^*P*<0.05 versus GFP treated with ISO, *n*=4 independent experiments. Images representative of four independent experiments are shown.

The above results indicate that SIRT3 in the nuclear of cardiomyocytes is abundantly expressed under ISO stimulation and SIRT3 overexpression, which makes it possible for SIRT3 to interact with PARP-1. To further illustrate the relationship between SIRT3 and PARP-1, we overexpressed SIRT3 by Ad-SIRT3 and used co-immunoprecipitation (CO-IP) to detect their interaction. The results of CO-IP showed that SIRT3 interacts with PARP-1 (Figure 7A). Since SIRT3 is a deacetylase and PARP-1 acetylation significantly enhances its activity, we simultaneously tested the level of acetylation of PARP-1. The results showed that PARP-1 acetylation was significantly enhanced by ISO, while overexpression of SIRT3 significantly inhibited the acetylation level of PARP-1 (Figure 7A). These results suggest that SIRT3 inhibit the activity of PARP-1 by inhibiting the acetylation of PARP-1.

In addition, we also studied the interaction between endogenous SIRT3 and PARP-1. Since SIRT3 is mainly localized in the cytoplasm but PARP-1 is mainly localized in the nuclear, we thus extracted the nuclear protein after 24 h stimulation with 10 μM ISO and detected the interaction between SIRT3 and PARP-1 by CO-IP. The results showed that the binding of SIRT3 to PARP-1 was significantly enhanced under ISO stimulation (Figure 7B).

**Figure 8:**
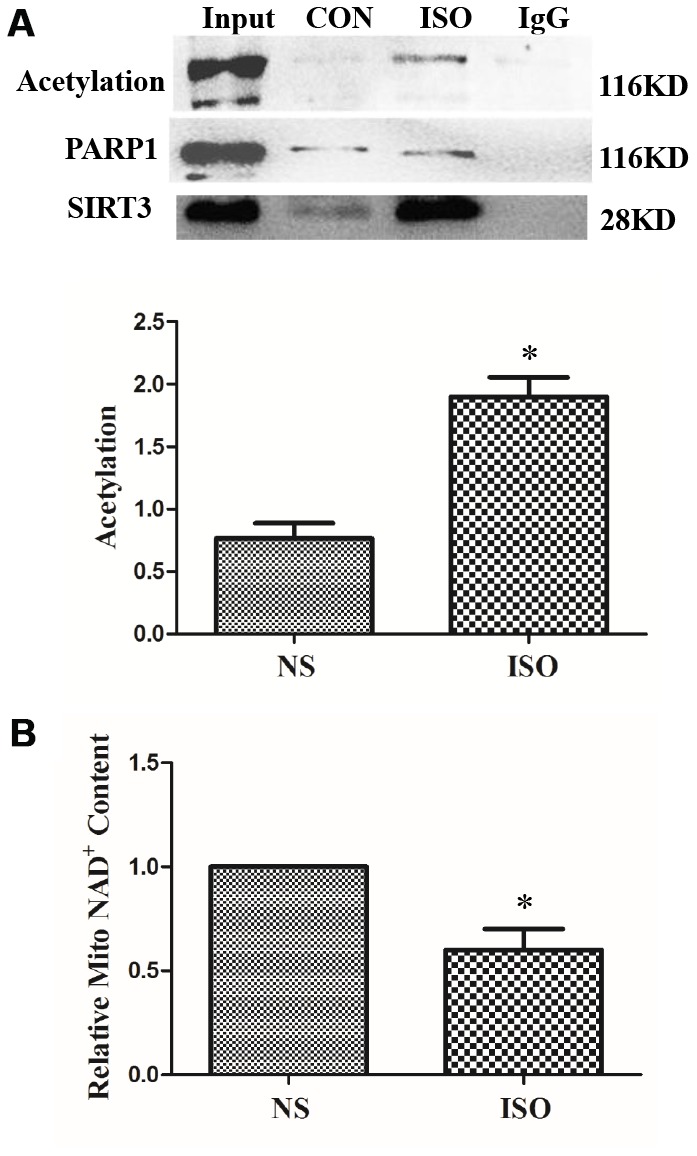
**SIRT3 interacts with PARP-1 in heart tissues**. SD rats of ISO group were subjected to subcutaneous injections of 1.5 mg/kg/d isoproterenol for 7 d. The control group (NS) was given the same dose of saline. Nuclear protein of heart tissues was extracted. PARP-1 was precipitated using PARP-1 antibody and detected with acetylated antibody, PARP-1 antibody, and SIRT3 antibody (**A**). NAD^+^ content in heart tissues of SD rats was detected by NAD^+^/NADH detection kit (**B**). Images representative of four independent experiments are shown. Data were presented as means±SE. *P<0.05 versus NS group, *n*=4 independent experiments.

In the ISO-induced *in vivo* cardiac hypertrophy model, nucleoproteins of heart tissues were extracted, and SIRT3 and PARP-1 interactions were also detected by CO-IP. The results showed that the PARP-1 acetylation level in the ISO group was increased, and the interaction between SIRT3 and PARP-1 was also significantly enhanced (Figure 8A).

In the cardiac hypertrophy model of ISO-stimulated SD rats, the interaction between SIRT3 and PARP-1 was enhanced, whereas the level of acetylation of PARP-1 was also increased. This may be due to the fact that ISO stimulation reduces the activity of SIRT3. Since SIRT3 is a type III histone deacetylase, its activity is dependent on intracellular NAD^+^ level, so we measured the level of NAD^+^ in cardiac tissue. The results showed that NAD^+^ was significantly reduced in the cardiac hypertrophy model of ISO-stimulated SD rats, suggesting that SIRT3 activity was reduced under ISO stimulation (Figure 8B).

## DISCUSSION

The protective role of SIRT3 in cardiac hypertrophy has been reported by several studies, but its main role is concentrated in the cytoplasm, and nuclear SIRT3 based anti-hypertrophic mechanism remains to be elucidated. Our study first showed that SIRT3 was up-regulated under ISO stimulation, and that adenovirus mediated overexpression of SIRT3 significantly inhibited the upregulation of ISO or PE-induced upregulated expression of cardiac hypertrophic genes-ANF and BNP (Figure 1). Studies have shown that long fragment of SIRT3 (44 KDa) is able to aggregate in the nuclear under stress conditions [4] (two of the human SIRT3 proteins: 44 KDa and 28 KDa, respectively, while the rat has only 28 KDa of SIRT3). Our results also showed that SIRT3 (28 KDa) expression was significantly enhanced in the nuclear under ISO stimulation (Figure 2). Furthermore, when SIRT3 was overexpressed with Ad-SIRT3, a distinct SIRT3 overexpression band was also detected in the nuclear (Figure 2). In addition, in primarily isolated neonatal rat cardiomyocytes, overexpressed with SIRT3-Flag or SIRT3-EGFP plasmids, SIRT3 expression were also detected in cytoplasm and nuclear (Supplementary Figure 2). These results suggest that nuclear SIRT3 may also play an important role in repressing cardiac hypertrophy. The results of heart tissue immunohistochemistry also showed that ISO stimulation increased the nuclear distribution of SIRT3 protein (Figure 3).

PARP-1, the most studied member of the PARP family, acts primarily as a DNA damage sensor in the nuclear and is activated in response to DNA single-strand breaks stimulated by free radicals and cell damage [36]. Poly (ADP-ribosylation) of PARP-1 requires NAD^+^ as a substrate. Overactivation of PARP-1 may deplete the storage of cellular NAD^+^, thereby reducing the function and activity of NAD^+^-dependent enzymes, such as the SIRTs family [36–39]. Thus, by direct ribosylation to modify its substrates, such as NF-κB, and to inhibit the function of NAD^+^ dependent enzymes, PARP-1 is involved in a variety of cardiovascular diseases, including heart failure, myocardial ischemia/reperfusion injury, atherosclerosis and cardiovascular complications of diabetes [40–42].

There are two methods for detecting PARP-1 activity: one is to use the anti-PAR-monoclonal antibody (that is, the method used in our study), which is a commonly used detection method, and this method is used in many high-quality articles [41, 43, 44]. When the signal detected by the PAR antibody is significantly enhanced, it means that PARP-1 activity is significantly enhanced, and more NAD^+^ is consumed [43, 44]. The second method was determined by a chemical quantitation method as described by Putt and Hergenrother (2004) [45], which converted NAD^+^ into a highly fluorescent agent using recombinant human PARP-1 (Sigma). The fluorescence intensity was determined by Multimode Microplate Reader (Infinite M1000, Tecan, Switzerland) at 360 nm for excitation and 445 nm for emission. To measure the cellular PARP-1 activity, the recombinant PARP-1 was replaced by nuclear extracts from cardiomyocytes and heart tissues. Because some cell experiments are performed in the presence of SIRT3 overexpression, SIRT3 requires NAD^+^ for its activity. Therefore, the second PARP-1 activity detection method is not suitable for detecting PARP-1 activity in this case.

Cardiac hypertrophy is an important stage of heart failure development, and PARP-1 has been shown to be critical for the development of cardiac hypertrophy. PARP-1 activity was found to be significantly upregulated in various animal models of pathological cardiac hypertrophy [21, 46], and PARP-1 deficient mice significantly attenuated AngII-mediated cardiac hypertrophy [41]. Likewise, PARP-1 inhibitor L-2286 significantly attenuated cardiac hypertrophy induced by pressure overload [21]. Another PARP-1 inhibitor AG-690/11026014 (6014), which was virtually screened, inhibited AngII-induced cardiomyocyte hypertrophy [47]. We also previously demonstrated that PARP-1 siRNA interference or the PARP-1 inhibitor 3-aminobenzamide (3-AB) also inhibits ISO-induced cardiomyocyte hypertrophy [19]. In addition, our previous studies have shown that ISO stimulation in neonatal rat cardiomyocytes significantly enhances the acetylation and activity of PARP-1 [19]. Consistent with these studies, we found significant activation of PARP-1 in cardiac hypertrophy induced by ISO or PE, and its level of acetylation was significantly elevated under the induction of ISO (Figures 5, 7A and 8A).

Since SIRT1 is a direct deacetylase of PARP-1, SIRT6 also interacts with PARP-1. Our study reveals SIRT3 as the third SIRTs member that directly interacts with and deacetylates PARP-1. After overexpressing SIRT3 by Ad-SIRT3, we detected the interaction between SIRT3 and PARP-1 by CO-IP. The results show that overexpression of SIRT3 can interact with PARP-1, and overexpression of SIRT3 can inhibit ISO-induced levels of PARP-1 acetylation (Figure 7A). In addition, under the action of ISO, the nuclear expression of SIRT3 was significantly enhanced, and correspondingly, the interaction between SIRT3 and PARP-1 was also significantly enhanced (Figures 7B and 8A).

We did not perform a loss of function test because it failed to block SIRT3 expression in the nuclear alone. Nevertheless, our overexpression experiments confirmed that SIRT3 overexpression can inhibit the acetylation and activity of the nuclear protein PARP-1, which shows that SIRT3 can exert its deacetylation effect in the nuclear (Figure 7A).

Taken together, our study reveals that SIRT3 exerts protective effects against cardiomyocyte hypertrophy by deacetylating PARP-1 and inhibit PARP-1 activity. These results will provide important clues for the study of the role of SIRT3 in cardioprotection and highlights the potential of SIRT3 activators in treating cardiovascular disorders.

## MATERIALS AND METHODS

Isoproterenol (ISO) was purchased from EMD Chemicals (San Diego, CA, USA). Phenylephrine (PE, P6126) were obtained from Sigma–Aldrich (Sigma, St. Louis, MO, USA). FBS and DMEM were purchased from Gibco (Grand Island, NY, USA). Ad-SIRT3 (due to the Flag label, its molecular weight is about 29 KDa) was purchased from Vigene Bioscience (Shandong, China). NAD^+^/NADH detection kit was purchased from Beyotime (Shanghai, China).

### Cell culture

H9c2 cardiomyocytes derived from rat ventricular myocardium were purchased from the Cell Bank of the Chinese Academy of Science (Beijing, China). Briefly, H9c2 cells were cultured in high glucose DMEM (GIBCO, USA) supplemented with 10% fetal bovine serum (GIBCO, USA) and 1% penicillin/streptomycin (GIBCO, USA) at 37°C in a humidified atmosphere with 5% CO_2_ [48].

### Animals

Sixteen male Sprague-Dawley (SD) rats (180–220g,) were supplied by the Experimental Animal Center of Anhui Medical University (Hefei, China). All animal care and experimental procedures were performed according to the Guide for the Care and Use of Laboratory Animals published by the US National Institutes of Health (NIH Publication No. 85-23, revised 1996) and were approved by the Animal Ethic Committee of Anhui Medical University. Cardiac hypertrophy was induced by *s.c.* injection of isoprenaline (1.5 mg/kg/d) for 7 consecutive days. Rats given normal saline (NS) were regarded as vehicle control group. The total number of rats was 8 per group. After 7 days, two-dimensionally guided M-mode echocardiography was performed using a VisualSonics Vevo 2100 system (VisualSonics, Toronto, ON) with a MS250 (21-MHz centerline frequency) probe. After assessment of echocardiography, rats were killed by exposing to a rising concentration of CO_2_. The hearts were carefully excised, and heart weight (HW) was determined. For morphometric measures, transverse sections of the hearts were fixed with neutral buffered formalin (10%), embedded in paraffin, cut into 5μm cross-sections and stained with haematoxylin and eosin (H&E).

### RNA isolation and quantitative RT-PCR (qRT-PCR)

Total RNA from cultured H9c2 cells was extracted using Trizol Reagent (Invitrogen, Carlsbad, CA, USA) following the manufacturer’s instructions [49]. One microgram of total RNA was reversely transcribed to first strand cDNA using One-step RT kit (Toyobo, Osaka, Japan). The mRNA levels of targeted genes were determined using Quantitative PCR kit (Toyobo, Osaka, Japan) by ABI 7500 system (Applied Biosystems, USA). Rat-specific primers for atrial natriuretic factor (ANF), brain natriuretic peptide (BNP), GAPDH were shown in Supporting Information Supplementary Table 1. GAPDH served as the endogenous control.

### Western blot and co-immunoprecipitation (co-IP)

For immunoprecipitation and Western blotting [50], mouse anti-PARP-1 polyclonal antibody, mouse pan-Acetylation monoclonal antibody and mouse β-Actin monoclonal antibody was purchased from Proteintech (Proteintech Group, Chi cago, IL, USA). Mouse anti-PAR-monoclonal antibody was purchased from Trevigen (Trevigen Inc., Gaithersburg, Maryland, USA). Rabbit anti-sirtuin 3 (SIRT3) polyclonal antibody were purchased from Cell Signaling Technology (Beverly, MA, USA) and Proteintech (Proteintech Group, Chi cago, IL, USA). Nuclear proteins were extracted with a commercially available Nuclear and Cytoplasm Extraction kit (Active Motif, Carlsbad, CA, USA) according to the manufacturer’s recommendations. Western blot analyses were performed as previously described [19, 51] and β-Actin was used as a loading control. For co-IP, total proteins (400μg) incubated with 1μg anti-PARP-1 antibody for overnight (mouse normal IgG was used as a control), or nuclear proteins (200-300μg) incubated with 1 μg anti-SIRT3 antibody for overnight (rabbit normal IgG was used as a control), followed by 4h incubation with protein A/G PLUS-Agarose (Santa Cruz, CA, USA) at 4°C. The co-IP proteins were detected by Western blot.

### Data analysis

Data are presented as mean±SE. Statistical analyses between two groups were performed by unpaired Student’s *t*-test. Differences among multiple groups were tested by one-way ANOVA with Tukey’s *post hoc* test. In all cases, differences were considered statistically significant with *P*<0.05.

## Supplementary Material

Supplementary Figures

Supplementary Tables
