# Supplementary material for: SIRT3 inhibits cardiac hypertrophy by regulating PARP-1 activity
Source: Aging (Albany NY). 2020 Mar 4;12(5):4178–92. doi: 10.18632/aging.102862 (PMC7093179; doi:10.18632/aging.102862)
Supplement: Supplementary Figures [file aging-12-102862-s002..pdf]

SUPPLEMENTARY FIGURES

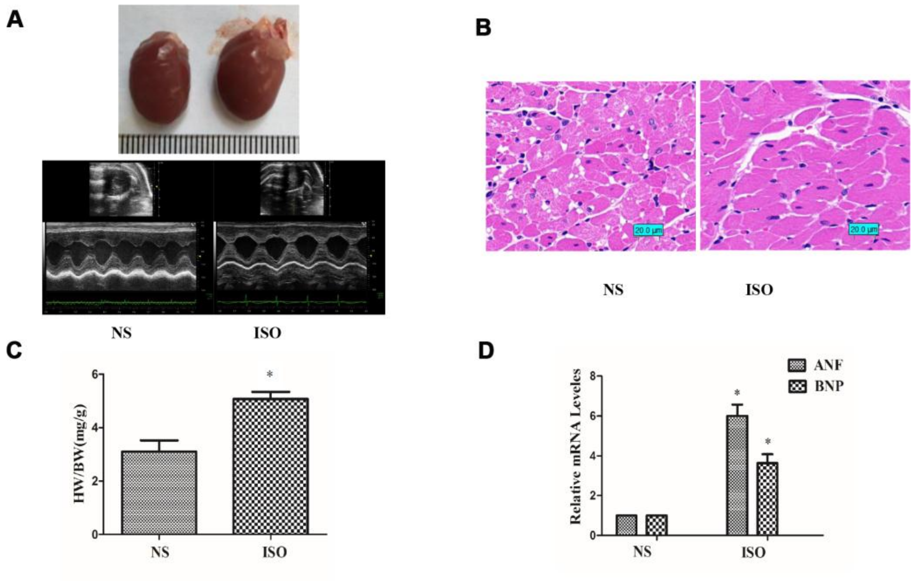

Supplementary Figure 1. SD rats were subjected to subcutaneous injections of 1.5 mg/kg/d isoproterenol for 7 d. (**A** and **B**) Pathological changes were observed with echocardiography and a light microscope at 400×magnification after H&E staining. (**C** and **D**) The ratios of the heart weight to body weight (HW/BW) and mRNA expression of ANF and BNP were calculated. Data were presented as means±SE. **P* < 0.05 *vs*. NS, *n* =6 (independent experiments).

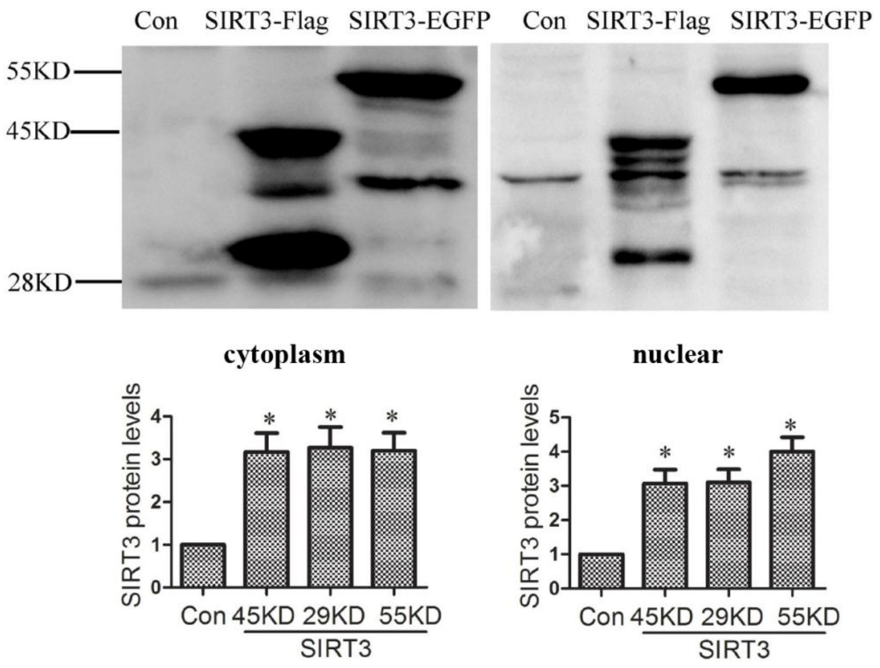

Supplementary Figure 2. The distribution of SIRT3 overexpression in primary neonatal rat cardiomyocytes. Cells were transfected with plasmids for SIRT3-Flag or SIRT3-EGFP. The protein expression levels of SIRT3 in cytoplasm and nuclear were measured by Western blot. Data were presented as means±SE. **P*<0.05 *vs.* Con group, *n*=3.
