# Supplementary material for: SIRT3 inhibits cardiac hypertrophy by regulating PARP-1 activity
Source: Aging (Albany NY). 2020 Mar 4;12(5):4178–92. doi: 10.18632/aging.102862 (PMC7093179; doi:10.18632/aging.102862)
Supplement: Supplementary Tables [file aging-12-102862-s001..pdf]

SUPPLEMENTARY TABLES

Supplementary Table 1. Primer sequences for quantitative RT-PCR.

Primer	Sequences
ANF	5'-CCTGGACTGGGGAAGTCAAC-3' (forward)
	5'-GTCAATCCTACCCCCGAAGC-3' (reverse)
BNP	5'-CAGAAGCTGCTGGAGCTGATA-3' (forward)
	5'-TCCGGTCTATCTTCTGCCCA-3' (reverse)
GAPDH	5'-GCGAGATCCCGCTAACATCA-3' (forward)
	5'-CTCGTGGTTCACACCCATCA-3' (reverse)

Supplementary Table 2. Gravimetric parameters of SD rats treated with ISO.

	NS(*n*=6)	ISO(*n*=6)
LVPW;d(mm)	1.796±0.403	2.533±0.629*
LVPW;s(mm)	1.737±0.286	3.500±0.703*
LVAW;d(mm)	1.737±0.286	2.155+0.448*
LVAW;s(mm)	2.510±0.330	3.199±0.526*

LVPW;d: left ventricular posterior wall depth at end-diastole; LVPW;s: left ventricular posterior wall depth at end-systole; LVAW;d: left ventricular anterior wall thickness during end-diastole; LVAW;s: left ventricular anterior wall thickness during end-systole; Data were presented as means±SE. *$P < 0.05$ *vs*. NS group.
